# Comparison of Fecal Microbiota in Children with Autism Spectrum Disorders and Neurotypical Siblings in the Simons Simplex Collection

**DOI:** 10.1371/journal.pone.0137725

**Published:** 2015-10-01

**Authors:** Joshua S. Son, Ling J. Zheng, Leahana M. Rowehl, Xinyu Tian, Yuanhao Zhang, Wei Zhu, Leighann Litcher-Kelly, Kenneth D. Gadow, Grace Gathungu, Charles E. Robertson, Diana Ir, Daniel N. Frank, Ellen Li

**Affiliations:** 1 Department of Medicine, Stony Brook University, Stony Brook, NY, United States of America; 2 Department of Applied Mathematics and Statistics, Stony Brook University, Stony Brook, NY, United States of America; 3 Department of Psychiatry, Stony Brook University, Stony Brook, NY, United States of America; 4 Department of Pediatrics, Stony Brook University, Stony Brook, NY, United States of America; 5 Department of Medicine, University of Colorado Anschutz Medical Campus, Aurora, CO, United States of America; Charité, Campus Benjamin Franklin, GERMANY

## Abstract

In order to assess potential associations between autism spectrum disorder (ASD) phenotype, functional GI disorders and fecal microbiota, we recruited simplex families, which had only a single ASD proband and neurotypical (NT) siblings, through the Simons Simplex Community at the Interactive Autism Network (SSC@IAN). Fecal samples and metadata related to functional GI disorders and diet were collected from ASD probands and NT siblings of ASD probands (age 7–14). Functional gastrointestinal disorders (FGID) were assessed using the parent-completed ROME III questionnaire for pediatric FGIDs, and problem behaviors were assessed using the Child Behavior Check List (CBCL). Targeted quantitative polymerase chain reaction (qPCR) assays were conducted on selected taxa implicated in ASD, including *Sutterella spp*., *Bacteroidetes spp*. and *Prevotella spp*. Illumina sequencing of the V1V2 and the V1V3 regions of the bacterial 16S rRNA genes from fecal DNA was performed to an average depth of 208,000 and 107,000 high-quality reads respectively. Twenty-five of 59 ASD children and 13 of 44 NT siblings met ROME III criteria for at least one FGID. Functional constipation was more prevalent in ASD (17 of 59) compared to NT siblings (6 of 44, P = 0.035). The mean CBCL scores in NT siblings with FGID, ASD children with FGID and ASD without FGID were comparably higher (58–62 vs. 44, P < 0.0001) when compared to NT children without FGID. There was no significant difference in macronutrient intake between ASD and NT siblings. There was no significant difference in ASD severity scores between ASD children with and without FGID. No significant difference in diversity or overall microbial composition was detected between ASD children with NT siblings. Exploratory analysis of the 16S rRNA sequencing data, however, identified several low abundance taxa binned at the genus level that were associated with ASD and/or first order ASD*FGID interactions (FDR <0.1).

## Introduction

Autism spectrum disorders (ASD) are neurodevelopmental disorders characterized primarily by alterations in reciprocal social interactions and communication as well as repetitive behavior. The reported prevalence of ASD has increased and affects 1 in 68 children in the USA [1]. This may be due in part to heightened awareness for these disorders [2,3]. The diagnosis of ASD is based on specially trained physicians and psychologists administering autism-specific behavioral evaluations. Twin studies provide support for both genetic and environmental contributions to ASD pathogenesis [2,3].

Alterations in the composition of resident gut microbial communities have been reported for an increasing number of human disorders, including ASD and functional GI disorders (FGID) [4–24]. The term “functional” in FGIDs refers to the absence of structural abnormalities in the gut that can be identified by blood, radiographic or endoscopic tests [25,26]. Consequently, FGIDs are diagnosed based on assessment of GI symptoms, and the exclusion of other GI diseases (e.g. celiac disease, inflammatory bowel diseases). FGIDs are very common in the general population and comprise a major portion of gastroenterology and primary care practices. Abnormalities in the neural regulation of gut function and sensation are implicated in the pathogenesis of FGIDs [20,25,26].

Functional GI symptoms have been frequently reported in ASD children as a non-neurological co-morbidity [27–31]. However a major barrier to integrating the ASD studies on GI symptoms with those conducted in the general population has been that the instruments used to assess functional GI symptoms in ASD differed from the ROME III process, which is the current international standard by which functional GI disorders (FGID) are diagnosed and classified [24].

In 2010, the Simons Foundation Autism Research Initiative (SFARI) began assembling the Simons Simplex Collection (SSC) as a resource for ASD research [32]. The SSC includes over 2000 simplex families, each with a single ASD proband but with unaffected parents and siblings. The simplex families were recruited through 12 university affiliated research clinics located in Canada and the United States. Upon recruitment to the SSC, the ASD probands (age 4–18) were evaluated with an extensive battery of diagnostic measures including the Autism Diagnostic Observation Schedule (ADOS) using the same diagnostic criteria across centers. Medical data, including GI symptoms, were collected on the ASD probands, but not on the NT siblings, using an extensive SSC Medical History form. Families were excluded if the proband did not meet criteria for ASD, had medically significant perinatal incidents (e.g. perinatal asphyxia), low mental age, or had primary relatives on the autism spectrum. The SSC thus represents a “unique, well-described sample of able children and adolescents with relatively severe ASD, as indicated by ADI-R and ADOS Calibrated Severity Scores (ADOS-CSS)” [32]. In order to assess the associations between ASD phenotype, FGIDs and fecal microbiota, we recruited a subset of the SSC families with ASD probands and/or NT siblings (age 7–14), to participate in a study collecting fecal samples and metadata related to functional GI disorders and diet.

## Materials and Methods

### Patient recruitment

Families were recruited via a registry called the Simons Simplex Community through the Interactive Autism Network (SSC@IAN), which is composed of simplex families originally recruited to the Simons Simplex Collection that were willing to be contacted through the Interactive Autism Network for additional studies. The SSC@IAN provided us with contact information for 245 potentially eligible simplex families that expressed their willingness to be contacted about this study. Of the 245 potentially eligible SSC families with 339 children, 107 families with 145 children consented to participate with parental written consents and written child assents for all participating children, and 66 families with 103 children (66 ASD probands and 37 NT siblings) completed the study. Exclusion criteria for ASD probands and NT siblings in this study were age <7 or age >14 years, a past or current history of GI diseases or other serious medical problems, and use of antibiotics within one month of collection of the stool sample. The procedures of the present study were reviewed and approved by the Institutional Review Board of Stony Brook University (IRB 313466)

Families who consented to participate in the study were asked to complete the Pediatric ROME III Version (QPGS-RIII), the GI symptoms form in the Simon Simplex Collection Medical History (v 4.0) [31], the Child Behavior Checklist (CBCL/6-18) [33–37], a one-week food diary for the ASD proband and/or the NT sibling prior to collecting a fecal sample, and provide current information on the child’s height and weight. The families were asked to submit the stool samples after the subjects were off probiotics and antibiotics for at least one month.

### Pediatric ROME III process of diagnosing FGID and the GI symptoms section of the SSC medical history form

Functional GI disorders (FGID) were assessed using the ROME III process [25]. Information on GI symptoms in both ASD and NT siblings was also collected using the GI symptoms section of the SSC Medical History form [32]. The GI symptoms section of the SSC Medical History form included the seven symptom categories: constipation, diarrhea, abnormal stool smell, bloating, excessive gas, severe abdominal pain, and vomiting. The parents were asked to rank whether these categories were problematic as “yes”, “not sure” and “no”. Many of these symptom categories in the SSC form overlap with the following symptoms included in the calculation of the GI severity index score [27]: constipation, diarrhea, stool smell, flatulence and abdominal pain, but did not include “unexplained daytime irritability”, “nighttime awakening” or “abdominal tenderness during exam”.

### Child Behavior Checklist (CBCL)

The CBCL/6-18 is a parent report form to screen for emotional, behavioral, and social problems in children ages 6–18 [33]. This instrument as well as the earlier version of this instrument has been previously used by several research groups studying school-aged children with ASD [34–37]. The data from ASD and NT siblings was entered and analyzed using the AGES 6–18 ASEBA-PC MODULE (ASEBA) to yield norm-referenced T-scores (M = 50, SD = 10). The CBCL contains eight syndrome scales: Anxious/Depressed, Withdrawn/Depressed, Somatic Complaints, Rule Breaking Behavior, Aggressive Behavior. The Internalizing Domain score is a measure of emotional problems and contains three syndrome scales: Anxious/Depressed, Withdrawn/Depressed, and Somatic Complaints. The Externalizing Domain score is a measure of behavioral problems and contains the Rule Breaking Behavior and Aggressive Behavior syndrome scales.

### Dietary information

The parents were asked to record the daily dietary intake of ASD and NT siblings on the Stony Brook University Medical Center Department of Pediatrics Food Diary/Calorie Count sheet for 7 days prior to the collection of the stool sample. The data were entered and analyzed using Nutritionist Pro nutrient analysis software version 5.2.0 (Axxya Systems, Nutritionist Pro, Stafford, TX). We focused on analyzing the following six macronutrient categories: total kcal/day, total protein g/day, total carbohydrates g/day, total fat g/day, total dietary fiber/day, total sugar g /day.

### Fecal sample collection and DNA extraction

Fecal samples were collected in a stool “hat” placed in the toilet and following evacuation 2 ml of stool were immediately transferred, using the spoon built into the lid into a Para-Pak Clean vial (Meridian Bioscience, Cincinnati, OH) that contained 10 ml of RNAlater for metagenomics studies and a Para-Pak Clean vial with no preservative. The fecal samples were shipped in cold packs overnight to our laboratory. Fecal DNA was extracted from stool samples immediately upon arrival using ZR Fecal DNA MiniPre (Zymo Research Corporation, Irvine, CA) and following manufacturer’s protocol. Fecal DNA and stool samples were stored in -80°C.

### Quantitative PCR (qPCR) for targeted bacterial subgroups

QPCR assays were performed using established primers for total bacteria (forward, 5’- GTG STG CAY GGY TGT CGT CA-3’ and reverse 5’- ACG TCR TCC MCA CCT TCC TC-3’) [38], *Sutterella* subgroup *(*forward, 5’-CGC GAA AAA CCT TAC CTA GCC-3’ and reverse 5’- GAC GTG TGA GGC CCT AGC C-3’) [10], *Bacteroidetes* subgroup (forward 5'-AACGCTAGCTACAGGCTT-3’ and reverse 5'-CCAATGTGGGGGACCTTC-3’) [39]; *Prevotella* (forward, 5’-CACCAAGGCGACGATCA-3’ and reverse 5’-GGATAACGCYGGACCT-3’) [40], *C*. *coccoides-E*. *rectales* subgroup (forward, 5’—CGGTACCTGACTAAGAAGC-3’and reverse 5’-AGTTT(C/T)ATTCTTGCGAACG-3’) [41], *Faecalibacterium prausnitzii* (forward 5’-CCC TTC AGT GCC GCA GT-3’ and reverse 5’-GTC GCA GGA TGT CAA GAC-3’) [42], *Escherichia coli* subgroup (forward, 5’- GTT AAT ACC TTT GCT CAT TGA-3’ and reverse, 5’- ACC AGG GTA TCT AAT CCT GTT-3’) [42]. The log_2_ transformation of the relative abundance of each bacterial subgroup was measured by ΔCt = Ct (threshold cycle) _total bacteria_−Ct _subgroup_. All assays were carried out in triplicate. Plasmid quantification standards were prepared from representative clones of the target organisms to insure that the assays were conducted within the linear range and with similar slopes.

### 16S rRNA amplicon library construction and Illumina V1V2 and V1V3 sequencing analysis

Fecal bacterial profiles were determined using broad-range amplification of the ~300 base pair (bp) V1V2 variable region using primers 27FYM (AGAGTTTGATYMTGGCTCAG) and 338R (TGCTGCCTCCCGTAGGAGT) and the ~500 bp V1V3 region using primers 27FYM (AGAGTTTGATYMTGGCTCAG) and 534R (ATTACCGCGGCKGCTGG) [43,44]. Sequencing was performed on the Illumina MiSeq platform using 600-cycle version 3 reagent kits. Demultiplexed, paired end sequence data were deposited in the NCBI Short Read Archive under BioProject Accession Number: PRJNA282013 (www.ncbi.nlm.nih.gov/bioproject/PRJNA2282203). The sorted paired reads were assembled using phrap [45] and paired reads that did not assemble were discarded. Assembled sequence ends were trimmed over a moving window of 5 nucleotides until average quality met or exceeded 20. Trimmed contigs with more than 1 ambiguity or shorter than 200 nucleotides were discarded. Potential chimeras identified with Uchime (usearch6.0.203_i86linux32) [46] using the Schloss [47] Silva reference sequences were removed from subsequent analyses. Assembled sequences were aligned and classified with SINA (1.2.11) using the 418,497 bacterial sequences in Silva 115NR99 as reference configured to yield the Silva taxonomy [48,49]. Operational taxonomic units (OTUs) were produced by clustering sequences with identical taxonomic assignments. OTU counts were normalized between samples by dividing sequence counts by the total number of sequences generated per sample to calculate the relative abundance. Phylum-level OTU tables were generated by collapsing lower level OTUs into higher-level categories. OTUs with a maximal relative abundance <0.0001 and with a prevalence <0.01 were culled. The remaining OTU relative abundances were then transformed using the square root function.

### Statistical analysis

Comparisons of gender, FGIDs, and SSC GI symptoms between ASD and NT siblings were conducted using the unconditional Fisher exact test with one tail [50]. The threshold significance was set at P < 0.05.

The ASD and NT siblings were further subdivided with respect to whether they also carried a diagnosis of an FGID based on the ROME III process. Study characteristics including body mass index (BMI), CBCL scores, dietary scores and the qPCR ΔCt scores were compared between the following four groups: 1) ASD with FGID, 2) ASD without FGID, 3) NT siblings with FGID and 4) NT siblings without FGID. Outcome variables including CBCL, dietary macronutrients, qPCR ΔCt were also modeled using mixed effect models with ASD, FGID and ASD*FGID as the three fixed effects, and family as random effects. Each continuous variable was fitted to a linear model while each categorical variable was fitted to a logistic model. Comparison of continuous variables between family matched ASD and NT siblings was conducted using two-tailed Student’s T test or the Wilcoxon signed rank test with the threshold of significance set as P < 0.05.

Alpha diversity indices (e.g. Chao1, Shannon complexity H, Shannon Evenness H/Ho) were calculated using Explicet [51] for each of the four groups with 10,000 replicate resamplings: 1) ASD with FGID, 2) ASD without FGID, 3) NT siblings with FGID and 4) NT siblings without FGID.

Beta diversity was compared between ASD and NT siblings using the adonis function in the R vegan package as previously described [52,53] at the phyla, family and genus level. This function uses a non-parametric multivariate analysis of variance test (PERMANOVA) [54], and was applied using Bray-Curtis, Jaccard and Morisita-Horn indices as distance measurements [55]. Family (i.e. whether ASD or NT siblings belonged to the same family) was selected as a strata variable to restrict permutations as previously described [56]. Overall microbial composition binned as phyla, was also compared between family matched ASD and NT siblings using the permutation Hotelling T test [57].

Paired two class comparisons of the transformed (square root) relative abundances of individual OTUs (phyla and genera level) between ASD probands and NT siblings from the same family were conducted using the Wilcoxon signed rank test with the threshold set as FDR < 0.05.

Because over-dispersion is often observed in microbiome sequence count data [58], a negative binomial regression model was also used to assess the effect of the ASD phenotype, FGID phenotype and first order interactions between ASD and FGID (ASD*FGID) on each individual OTU, in both paired and unpaired ASD and NT samples as follows:

$$log\left(\mu_{ijk}\right)\;=\;{\left(\beta_{i0}\right)}_{k}+\beta_{1k}ASD_{ij}+\beta_{2k}FGID_{ij}+\beta_{3k}ASD\;*\;FGID_{ij}+log\left(Y_{ij.}\right)$$

$${\left(\beta_{i0}\right)}_{k}\;=\;b_{0k}+b_{ik}I\{{family}_{ij}\;=\;i\}$$

$$Y_{ijk}∼NB\left(\mu_{ijk},\phi_{k}\right).$$

where *Y*_*ijk*_ denotes the raw sequence count of OTU *k* for child *j* in family i. In this model we treat family, which may reflect genetic and possibly dietary factors, as a zero mean random coefficient. Coefficients *b*_0_, *β*_1_, *β*_2_ and *β*_3_ are fixed constants representing grand mean, the effect of ASD, FGID and the interaction of ASD and FGID respectively. The log total sequence count ($Y_{ij.}=\sum_{k}\;Y_{ijk}$) for each individual is considered as an offset. Because the distribution of the raw sequence count is often skewed by a large proportion of zero counts, a zero-inflated version of the negative binomial model was also fitted to each individual OTU: The final model was selected based on the Akaike Information Criterion (AIC). The p-values for each OTU were adjusted for multiple comparisons to calculate a false discovery rate (FDR) using the Benjamini-Hochberg procedure [59]. The threshold of significance for ASD, FGID or ASD*FGID first order interactions was set at FDR < 0.1. The logistic and linear mixed models were built with R package glmmADMB [60] and lme4-R [61] respectively.

Comparative analysis of the parallel square root transformed relative abundance [62] measurements of Bacteroidetes phylum, the Sutterella genus and the Prevotella genus by MiSeq V1V2, MiSeq V1V3 and targeted qPCR was conducted using latent structural equation modeling [63]. Because of the large proportion of zero counts for Sutterella and Provetella genera, samples with zero counts were excluded from the analysis. To avoid the complication caused by correlated samples, SEM models were fitted with grouping variable ASD and the constraint that loadings are equal for different groups. The corresponding model for each group is: The corresponding model for each group is:

$$qPCR_{ij}=a_{1}+b_{1}\xi_{ijk}+\delta_{1}$$

$$V1V2_{ij}=a_{2}+b_{2}\xi_{ijk}+\delta_{2}$$

$$V1V3_{ij}=a_{3}+b_{3}\xi_{ijk}+\delta_{3}$$

Here *ξ*_*ij*_ is the underlying true bacteria relative abundance in the square root scale, while *δ*_1_, *δ*_2_ and *δ*_3_ are the corresponding measurement errors. In order to render the model identifiable, we chose qPCR as the reference level with *a*_1_ = 0 and *b*_1_ = 1. The model was fitted with the R package lavaan [64].

## Results

### Demographics and functional GI disorders (FGID) in ASD and NT siblings

A total of 59 ASD children and 44 NT siblings from 66 families completed the study (**Table 1)**. Matched ASD and NT sibling pairs were from 37 families. In some instances NT siblings of ASD probands were recruited, but not the ASD proband s because their age exceeded 14 years. The ASD children were predominantly male. The gender distribution for the NT siblings was roughly equal. The mean age in both ASD children and NT siblings was 10 years, and they were predominantly of Caucasian descent. There are three major groups of FGIDs (see **Table 1**) [24]: H1) Vomiting and aerophagia, H2). Abdominal-pain related FGIDs, and H3) Constipation and fecal incontinence. Group H1 is further subdivided into three FGIDs: H1a) Adolescent rumination syndrome; H1b) Cyclic vomiting syndrome; H1c) Aerophagia. Group H2 is further subdivided into four FGIDS: H2a) Functional dyspepsia, H2b) Irritable bowel syndrome (IBS), H2c) abdominal migraine, H2d) Childhood functional abdominal pain with a subset termed H2d1. Childhood functional abdominal pain syndrome. Subjects classified within the H2d1 subset have additional somatic symptoms such as headache, limb pain or difficulty sleeping.

**Table 1:** Demographics and functional GI disorders (FGID) in ASD and NT siblings. The study characteristics of the ASD and NT siblings are listed below. For categorical variables, the percent are shown in parenthesis (). For continuous variables the mean ± standard deviations are listed.

Subject Characteristics	ASD n = 59	NT n = 44	P-value
**Family matched**	37	37	
Gender (Male)*	52 (88%)	21 (48%)	0
**Age (years)**	10.3 ± 1.8	10.0 ± 1.8	
**Race/Ethnicity**			
White/Non-Hispanic	49 (83%)	33 (75%)	0.16
Hispanic	2 (3%)	4 (9%)	0.13
Black	4 (7%)	3 (7%)	0.51
Other/Unknown	4 (7%)	4 (9%)	0.34
**FGID present (all patients)**	25 (42%)	13 (30%)	0.1
**FGID present (family matched)**	15 (41%)	12 (32%)	0.31
***H1*. *Vomiting and Aerophagia***			
H1a. Adolescent rumination syndrome	0	0	
H1b**.** Aerophagia	2	2	0.44
H1c. Cyclic Vomiting Syndrome	0	1	0.33
***H2*. *Abdominal-pain related FGIDs***			
H2a. Functional Dyspepsia	0	0	
H2b. Irritable Bowel Syndrome	7	3	0.21
H2c. Functional Abdominal Migraine	1	3	0.13
H2d. Childhood Functional Abdominal Pain	0	2	0.12
H2d1 Childhood Functional Abdominal Pain Syndrome	0	2	0.12
***H3*. *Constipation and fecal incontinence***			
Functional Constipation*	17	6	0.035
Nonretentive Fecal Incontinence	3	1	0.3

Twenty-five of 59 (42%) ASD children and 13 of 44 (30%) NT siblings were diagnosed with at least one FGID. Fifteen ASD children and 13 NT siblings in 37 families met ROME III criteria for at least one FGID, with concordance for FGIDs in 6 (18%) ASD/NT sibling pairs. Some ASD and NT siblings were diagnosed with more than one FGID. ASD children exhibited a higher prevalence of functional constipation in compared to NT siblings (P = 0.035) when both matched and unmatched individuals were included in the analysis. We detected no significant difference in the prevalence of children with at least one FGID in family matched ASD/NT sibling pairs. We also detected no significant difference in the prevalence of specific FGIDs such as functional constipation in the family matched ASD/NT sibling pairs.

When the SSC registry was established, medical history forms including one on GI symptoms were completed by the parents on the ASD children but not the NT siblings. The same SSC GI symptoms form was then completed (~ 2–3 years later) by the parents for the ASD and NT children for the current study. As shown in **Table 2**, there was no significant change in the prevalence of the GI symptoms occurred over time. Furthermore, the SSC results indicate that ASD children exhibited a higher prevalence of constipation compared to NT siblings (P = 0.05).

**Table 2:** SSC problematic GI symptoms results. The previous and current percent of ASD children and NT siblings having a problematic GI symptom is listed below. The previous percent were based on parental responses to the SSC medical form for ASD children only on first entering the SSC registry. The current percent were based on parental responses to the same forms for both ASD and NT siblings during the current study ~2–3 years later. The P-values reflect comparison of the current responses of the ASD and NT siblings. There was no significant change in the prevalence of any of the seven problematic GI symptoms in the ASD children at the two time points.

Problematic GI Symptoms	ASD n = 59		NT n = 44	P-value ASD vs. NT
	Previous	Current	Current	Current
**Excessive Gas**	7%	5%	2%	0.32
**Vomiting**	7%	3%	0%	0.33
**Bloating**	3%	2%	2%	0.49
**Severe Abdominal Pain**	0%	0%	7%	0.07
**Constipation**	31%	32%	18%	0.05
**Diarrhea**	14%	11%	5%	0.24
**Smelly Stools**	7%	7%	0%	0.33

### Comparison of CBCL and macronutrient intake

As shown in **Table 3**and **S1 Table**, the ASD severity score, ADOS-CSS, did not differ significantly between the ASD w FGID group and the ASD w/o FGID group. Mean total, internalizing, externalizing and subscale CBCL scores were significantly increased (p < 0.05) in the ASD children in both the ASD/NT sibling pairs and in comparison of all the ASD with NT children (matched and unmatched). These CBCL scores were also significantly increased (p < 0.05) in NT siblings with FGID compared to NT siblings without FGID (**Table 3**). There was no significant difference in the mean CBCL scores between ASD children with FGID compared with ASD children without FGID.

**Table 3:** Comparison of ADOS-CSS, CBCL scores and macronutrient intake between ASD children with FGID, ASD children without FGID, NT siblings with FGID and NT siblings without FGID. For continuous variables the mean values ± standard deviations are listed. N.A., not available.

Study Characteristics	ASD w FGID N = 25	ASD w/o FGID N = 34	NT w FGID N = 13	NT w/o FGID N = 31
**ADOS-CSS**	7.7 ± 1.6	7.6 ± 1.7	N.A.	N.A.-
**IQ**	95.8 ± 18.2	86.6 ± 26.2	N.A.	N.A.
**CBCL Total**	62.2 ± 7.3	58.8 ± 8.9	59.2 ± 10.3	43.6 ± 9.6
Internal	61.2 ± 8.8	57.6 ± 9.7	60.2 ± 7.8	46.2 ± 8.3
External	54.0 ± 9.9	52.4 ± 10.4	57.7 ± 10.9	44.6 ± 8.2
Anxious/Depressed	59.3 ± 7.4	58.4 ± 8.4	60.0 ± 8.7	52.7 ± 4.3
Withdrawn/Depressed	63.2 ± 9.8	60.5 ± 7.0	53.9 ± 3.8	51.9 ± 3.4
Somatic Complaints	58.7 ± 7.2	55.5 ± 6.3	63.2 ± 5.6	52.4 ± 4.1
Rule Breaking Behavior	54.8 ± 6.8	53.2 ± 4.1	58.2 ± 8.1	51.8 ± 3.4
Aggressive Behavior	57.3 ± 7.8	56.8 ± 7.5	60 ± 8.8	51.6 ± 3.1
Social Problems	62.4 ± 8.1	60.3 ± 7.5	55.5 ± 7.3	52.4 ± 4.1
Thought Problems	67.2 ± 7.6	63.5 ± 8.1	58.9 ± 9.0	52.4 ± 3.4
Attention Problems	65.8 ± 10.5	63.4 ± 8.4	59.5 ± 7.2	52.7 ± 3.7
**Diet Daily Total Kcal**	2090 ± 500	2040 ± 480	1870 ± 490	1880 ± 350
Daily Protein (g)	66 ± 21	69 ± 16	61 ± 18	69 ± 14
Daily Fat (g)	75 ± 20	76 ± 20	72 ± 25	70 ± 18
Daily Carbohydrate (g)	294 ± 81	277 ± 74	382 ± 424	277 ± 74
Daily Sugar (g)	123 ± 41	109 ± 37	117 ± 48	106 ± 34
Daily Dietary Fiber (g)	18 ± 7	18 ± 8	13 ± 5	16 ± 5
**Gluten or casein free**	1	3	0	1
**Body mass index (kg/m** ^**2**^ **)**	18.3 ± 4.1	18.4 ± 4.2	18.5 ± 4.0	17.7 ± 3.0

No significant differences (p < 0.05) between the four study groups with respect to daily intake of macronutrients (calories, protein, fats, carbohydrates, sugars or dietary fiber) were noted (**Table 3**). Only 4 children were reported to adhere to a gluten and/or casein free diet. The study participants had BMIs that were within the normal range and were not obese.

### No associations (FDR < 0.1) detected in targeted bacterial subgroups between ASD, FGID and/or first order ASD*FGID by QPCR

Targeted qPCR assays of fecal samples were conducted for *Sutterella*, *Prevotella* and total *Bacteroidetes* subgroups, because of previous reports that the relative abundance of these subgroups differed between ASD and NT children [7,8,10–14]. QPCR assays targeting the *C*. *coccoides-E-rectales* group, *Faecalibacterium prausnitzii* and *Escherichia coli* were also performed because altered relative abundances have been reported previously in GI disorders such as inflammatory bowel diseases [23]. The mean ΔCt values of these subgroups, which represent a log_2_ transformation of the relative abundance, for 1) ASD w FGID, 2) ASD w/o FGID, 3) NT w FGID and 4) NT w/o FGID are shown in **Table 4**. No significant difference was observed for any of these bacterial subgroups in the 37 ASD/NT sibling pairs (see Methods). Furthermore, in mixed models, no significant effect of ASD, FGID or ASD*FGID was observed in an analysis of all 59 ASD and 44 NT siblings.

**Table 4:** QPCR comparisons of selected bacterial subgroups in ASD children with FGID, ASD children without FGID, NT siblings with FGID and NT siblings without FGID. The qPCR assays were conducted using established primers as described in Methods. The mean ΔCt values (~Log2 relative abundance of targeted bacterial subgroups) ± standard deviations are listed. The data from family matched and unmatched ASD and NT siblings are included.

	ASD w FGID N = 25	ASD w/o FGID N = 34	NT w FGID N = 13	NT w/o FGID N = 31
***Sutterella* ΔCt**	-10.4 ± 2.1	-10.1 ± 2.8	-11.9 ± 3.8	-10.3 ± 3.1
***Prevotella* ΔCt**	-14.9 ± 4.5	-14.7 ± 4.7	-14.4 ± 4.9	-14.2 ± 4.3
***Bacteroidetes* ΔCt**	-3.1 ± 2.5	-2.1 ± 5.2	-3.4 ± 3.0	-2.2 ± 7.0
***C*. *coccoides*- *E*. *rectales* ΔCt**	-6.7 ± 4.5	-5.7 ± 3.0	-5.7 ± 2.4	-5.7 ± 2.2
***Faecalibacterium prausnitzii* ΔCt**	-6.5 ± 1.5	-6.2 ± 1.2	-7.0 ± 3.2	-6.0 ± 2.2
***Escherichia coli* ΔCt**	-18.4 ± 5.4	-16.7 ± 4.8	-15.8 ± 4.2	-17.6 ± 4.6

### 16S rRNA sequence analysis of ASD children and NT siblings

We performed Illumina sequencing of the V1V3 hypervariable region of the bacterial 16S rRNA gene to facilitate comparisons of this data with previous 16S rRNA 454 pyrosequencing datasets of the V1V3 hypervariable region [16,23]. A total of 11,050,770 high-quality sequences were generated (median 1.1E+05 sequences/sample, IQR 8.0e+04 to 1.2E+05). All libraries had a Good’s coverage score ≥ 99.9% at the rarefaction point of 15,000 sequences, indicating deep sequence coverage of the intestinal microbiome was achieved for each sample. To corroborate results generated through V1V3 sequencing, we also performed Illumina sequencing of the V1V2 hypervariable region. A total of 14,783,994 high-quality sequences were generated (median 1.4E+05 sequences/sample, IQR 1.3e+05 to 1.6E+05; minimum Good’s coverage = 99.9%). No significant effect of ASD, FGID or ASD*FGID was observed on OTU complexity or richness, two measures of α-diversity, Shannon diversity and Chao 1 (see **Table 5**for mean values). No significant difference (p < 0.05) was noted in β-diversity between ASD and NT sibling bacterial communities analyzed at the phyla, family or genus level and using Bray-Curtis, Jaccard or Morisita-Horn indices as dissimilarity measurements.

**Table 5:** Shannon H and SChao1 scores in ASD children with FGID, ASD children without FGID, NT siblings with FGID and NT siblings without FGID. The mean values for each of these scores ± standard deviation are shown below for the V1V2 and V1V3 datasets. The data from family matched and unmatched ASD children and NT siblings are included.

	V1V2		V1V3	
	Shannon H	SChao 1	Shannon H	SChao 1
**ASD w FGID**	3.53 ± 0.55	85 ± 14	3.60 ± 0.423	72 ± 10
**ASD w/o FGID**	3.41 ± 0.53	78 ± 9	3.45 ± 0.52	65 ± 9
**NT w FGID**	3.46 ± 0.57	90 ± 14	3.54 ± 0.48	70 ± 9
**NT w/o FGID**	3.42 ± 0.52	82 ± 14	3.38 ± 0.52	67 ± 13

**Fig 1:**
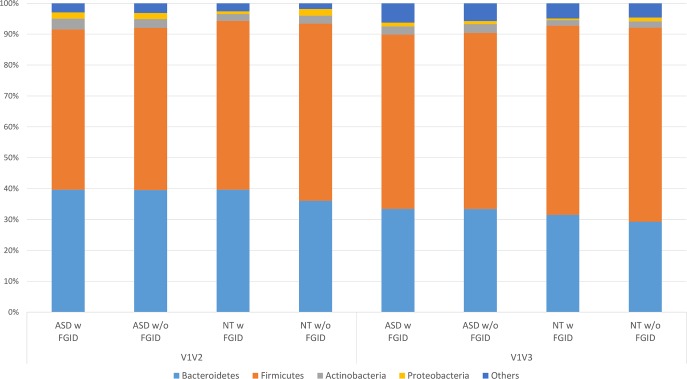
Phyla comparison between ASD children with FGID, ASD children without FGID, NT siblings with FGID and NT siblings without FGID, using Illumina V1-V2 and V1-V3 datasets. The average relative abundance of each phyla (relative abundance ≥ 0.02) is shown for ASD w FGID, ASD w/o FGID, NT w FGID and NT w/o FGID for each of the two sequencing datasets (See S1 and S2 Tables for means ± standard deviations).

The relative abundances of the major four phyla, (Firmicutes, Bacteroidetes, Actinobacteria, Proteobacteria) for the ASD children with FGID, ASD children without FGID, NT siblings with FGID and NT siblings without FGID groups for the V1V2 and V1V3 datasets are shown in **Fig 1**(see **S2 Table**). Phyla of lower abundance, including RF3, Tenericutes, Cyanobacteria, Verrucomicrobia, Lentisphaerae, and Fusobacterium were also detected (see **S3 Table**). Permutational Hotelling T test revealed no significant difference in the overall microbial composition when binned at the phyla level, between ASD children and NT siblings. With the exception of the Verrucomicrobia phylum, the relative abundances of the phyla were similar between the V1V2 and V1V3 data sets. Exploratory analyses of individual phyla detected no significant effect (FDR <0.1) of ASD, FGID or ASD*FGID first order interactions for any of the individual phyla, including Bacteroidetes, using a negative binomial model. At the genus level, 359 operational taxonomic units (OTU) were detected for the V1V2 dataset and 189 OTUs for the V1-V3 datasets, respectively. No significant associations were detected between the relative abundance of the Sutterella or Prevotella genera and ASD, FGID or ASD*FGID first order interactions (see **Table 6**) in either the V1V2 or V1V3 datasets. Exploratory analyses of individual genera revealed a significant effect (FDR < 0.1) of ASD and ASD*FGID first order interactions on the *Cyanobacteria/Chloroplast* genus for both the V1V2 and/or V1V3 datasets using a zero-inflated negative binomial model (see **Table 6).** The mean relative abundance of this genus was increased in the ASD-with FGID group compared to those of the other three categories (ASD without FGID, NT siblings with FGID and NT siblings without FGID, see **S4 Table** for mean relative abundances). 16S RNA sequence analyses of the human gut microbiota have previously detected sequences binned within the Cyanobacteria phylum. These sequences could represent chloroplast 16S rRNA genomic sequences present in dietary plant foods or novel uncultivated bacterial species. Metagenomic studies have detected a novel nonphotosynthetic taxa termed “Melainabacteria” present in human gut flora that represents either a sibling phylum or a class within the Cyanobacteria phylum [65,66]. The Silva database used in the current study, however, binned the Melainabacteria sequences in a taxon that was distinct from the *Chloroplast* taxon. Inspection of the daily food diaries of all the subjects revealed that two ASD children with FGID (functional constipation), who had high relative abundances of this genus and consequently dominated the signal associated with ASD with FGID category, were fed chia seeds added to smoothies. None of the other subjects consumed chia seeds except one ASD child who ingested a serving of mixed grain bread that included chia grain. Of note, chia seeds have been touted as an autism cure on the internet [67,68].

**Table 6:** Low abundance taxa binned at the genera level that are significantly associated with ASD, FGID or ASD*FGID first order interactions. The FDRs for each low abundance taxa are listed below for the V1V2 and V1V3 datasets, with the threshold of significant <0.1. N.D. is not detected based on filtering criteria. The FDRs are also listed for two genera, Sutterella and Provetella, which have been previously reported to be increased and decreased, respectively, in ASD children compared to unrelated control children [10,11].

	V1V2			V1V3		
Phylum/Taxa	ASD	FGID	ASD* FGID	ASD	FGID	ASD* FGID
***Cyanobacteria/Chloroplast***	**0.027**	0.228	**0.0005**	**0.0702**	0.280	**0.0026**
***Firmicutes/Asteroleplasma***	**0.000009**	0.228	**0.0006**	0.742	0.591	0.644
***Proteobacteria/Thalassospira***	**0.065**	**0.073**	**0.020**	0.609	0.591	0.640
***Proteobacteria/Burkholderia***	0.927	**0**	**0**	0.861	0.871	0.939
***Proteobacteria/Comamonadaceae***	0.945	1	0.959	**0.007**	0.177	**0.017**
***Fusobacteria*.*Fusobacteriales***	0.92	0.826	0.797	**0**	**0**	**0**
***Bacteroidetes/Prevotellaceae***	0.920	0.875	0.819	0.433	0.591	**0.033**
***Actinobacteria/Mobiluncus***	N.D.	N.D.	N.D.	**0.003**	0.591	**0.037**
*Proteobacteria/Sutterella*	0.928	0.905	0.824	0.999	0.999	0.999
*Bacteroidetes/Prevotella*	0.712	0.595	0.601	0.805	0.591	0.876

For the V1V2 dataset only, significant associations were observed for 1) ASD and ASD*FGID first order interactions with *Firmicutes/Asteroleplasma*, 2) ASD, FGID and ASD*FGID first order interactions with *Proteobacteria/Thalassospira*, and 3) FGID and ASD*FGID first order interactions with *Proteobacteria/Burkholderia* (see **Table 5**and **S4 Table**). The *Firmicutes/Asteroleplasma* genus appears to represent a group of uncultured wall-less mycobacterium like organisms within the class *Erysipelotrichia* [69]. The *Proteobacteria/Thalassospira* genus appears to represent alpha-proteobacteria present in bacterioplankton and a number of uncultivated organisms [70]. The sequences binned in this *Proteobacteria/Burkholderia* taxon represent unclassified sequences within the *Burkholderia* order that did not bin within the *Comamonadaceae* family (see below).

For the V1V3 dataset only, significant associations were observed for 1) ASD and ASD*FGID first order interactions with the *Proteobacteria/Comamonadaceae*, 2) ASD, FGID and ASD*FGID first order interactions with *Fusobacteria*.*Fusobacteriales*, 3) ASD*FGID first order interactions with *Bacteroidetes/Prevotellaceae*, and 4) ASD and ASD*FGID interactions with *Actinobacteria/Mobiluncus*. These associations, however, did not reach significance in the V1V2 dataset. *Proteobacteria/Comamonadaceae* is a large and diverse bacterial family within the *Burkholderiales* order (see above). The sequences binned within this *Fusobacteria/Fusobacteriales* taxon represent unclassified sequences within the *Fusobacteriales* family that did not bin within the *Fusobacterium* genus. The sequences binned within the *Bacteroidetes/Prevotellaceae* taxon represent unclassified sequences within the *Prevotellaceae* family that did not bin within the *Prevotella* genus. The *Actinobacteria/Mobiluncus* genus has previously been associated with bacterial vaginosis [71].

### Comparison of MiSeq V1V2, V1V3 and qPCR measurements of selected bacterial categories

Comparisons of the relative abundances of the Bacteroidetes phylum, and the *Sutterella* and *Prevotella* genera reveal high correlations between MiSeq V1V2 and V1V3 **(ρ≈**0.9) and small proportional biases **(S5 Table** and **S1 Fig).** The different reverse primers, 338R and 534R, used for amplifying respectively the V1V2 and V1V3 regions, might have subtle differences in specificity. While there were generally positively correlated measurements between the MiSeq and qPCR platforms, we observed large systematic biases particularly with the two relative low abundance genera, *Sutterella* and *Provetella*. This may reflect at least in part that the depth of sequencing was not sufficient to accurately measure relatively low abundance bacterial categories.

## Discussion

**Fig 2:**
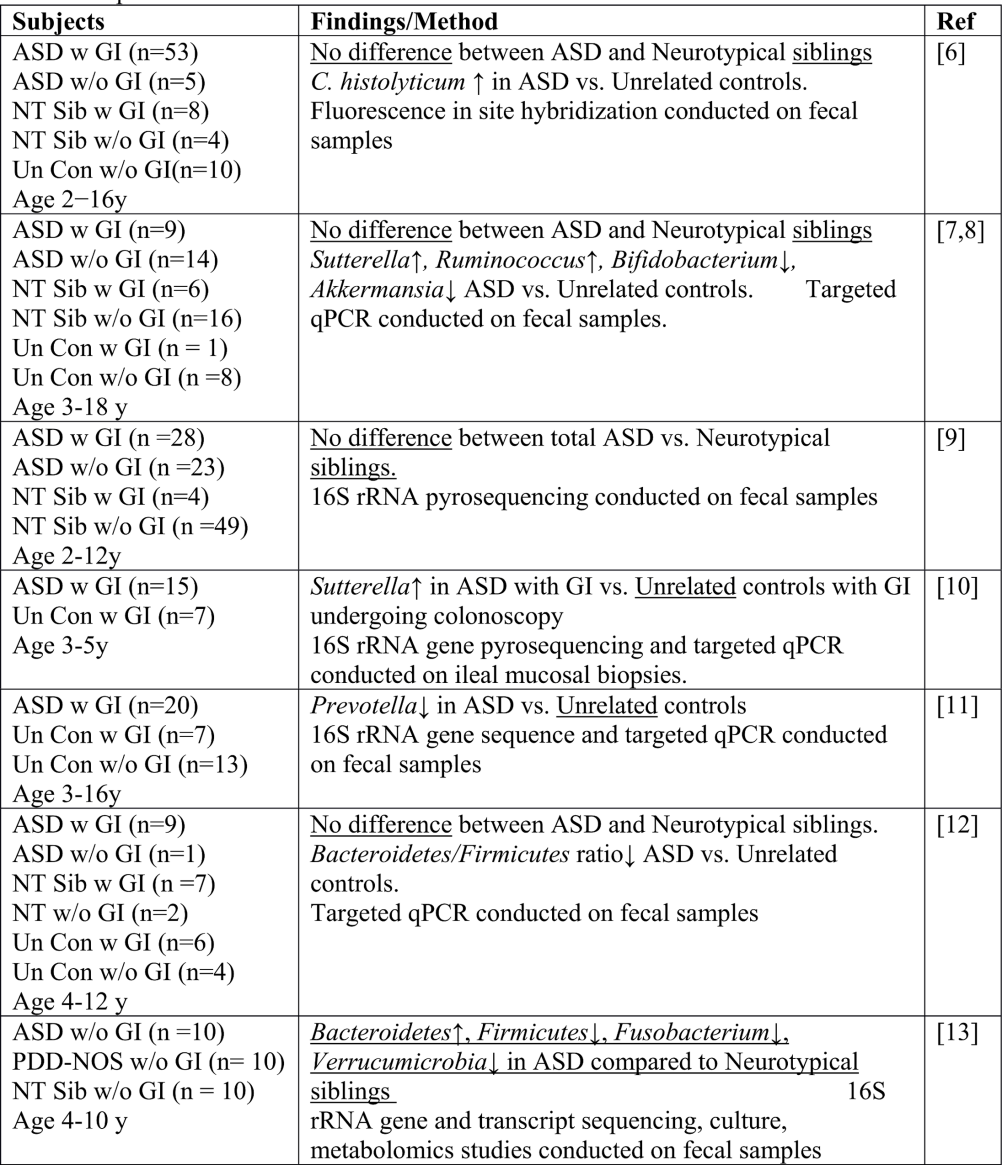
Summary of studies comparing gut microbiota in ASD and NT children with and without GI symptoms. ASD w GI, ASD children with GI symptoms, ASD w/o GI, ASD children without GI symptoms, NT Sib w GI, NT siblings with GI symptoms; NT sib w/o GI, NT siblings without GI symptoms, NT Un Con w GI, unrelated controls with GI symptoms; Un Con w/o GI, unrelated controls without GI symptoms; PDD-NoS, pervasive developmental disorder not otherwise specified.

**Fig 3:**
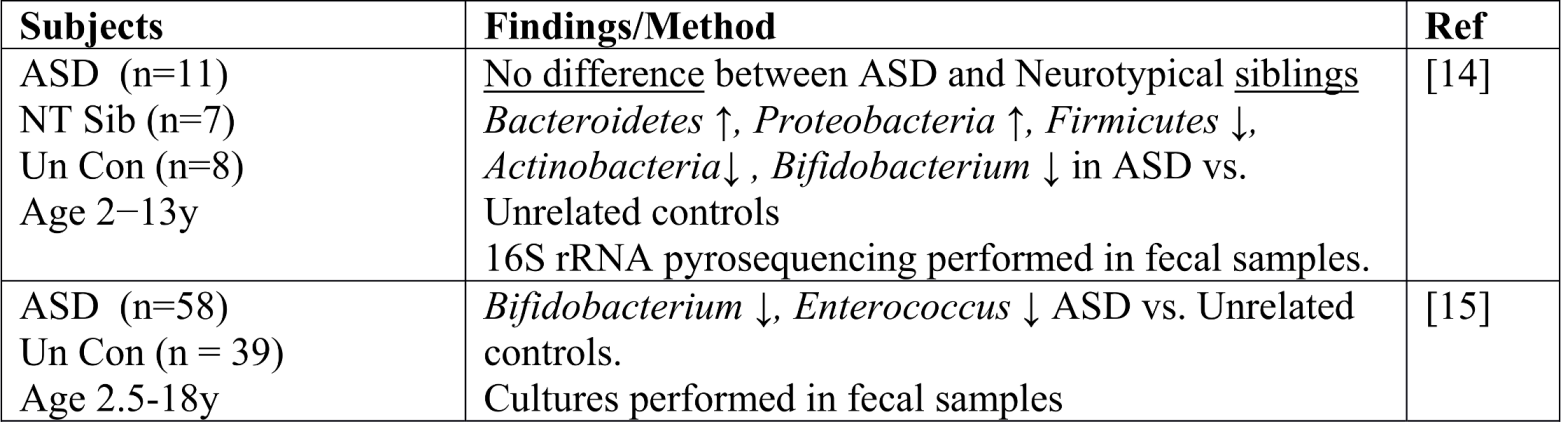
Summary of studies comparing gut microbiota in ASD and NT children with no information on GI symptoms. NT Sib, neurotypical sibling, Un Con, unrelated control.

This study of 59 ASD children and 44 NT siblings in the Simons Simplex Community through the Interactive Autism Network (SSC@IAN) registry, was motivated by previous reports linking ASD, GI symptoms with alterations in the gut microbiota (see **Fig 2)** and some simply linking ASD with the gut microbiota (see **Fig 3**)**.** These previous studies of gut microbial communities in ASD children used both unrelated control children and NT siblings of ASD children. One study also included related disorders, such as Aspergers or Pervasive developmental disorder not otherwise specified (PDD-NOS) as the non-ASD control population. Our current study compared microbial composition and diversity between ASD children with only NT siblings. Of the 44 NT siblings, 37 were family matched to the ASD children in the study and the remaining seven NT children were siblings of ASD children who were not in the current study, in some cases because the ASD child was older than 14 years of age. This study did not replicate previously reported differences in the relative abundances of bacterial categories, including the Bacteroidetes phylum [12–14], the Sutterella genus [7,8,10] and the Prevotella genus [11] between ASD and unrelated control children. This may reflect the smaller number of subjects enrolled in the previous studies, possibly resulting in Type 1 errors. One study used intestinal mucosal [10] rather than fecal samples. Of note, previous studies [6–8,12,14], with the exception of one study [13], failed to detect differences in microbial composition between ASD and NT siblings [11]. Our study also detected no differences in either α or β diversity between ASD and NT siblings.

In this study, 16S rRNA sequencing using MiSeq was carried out to a greater depth than previous studies utilizing pyrosequencing. Both platforms detected an increased relative abundance of the low abundance Chloroplast genus in ASD compared to NT siblings. Inspection of the daily dietary logs of the ASD children with high counts of Chloroplast sequences revealed that these particular children were fed shakes containing chia seeds. We speculate that the observed increase in the relative abundance of Chloroplast sequences could reflect ingestion of chia seeds by these children.

An alternative explanation for why a study comparing ASD children with NT siblings did not replicate differences reported between ASD children with unrelated controls, is that NT siblings of ASD children have altered microbiomes compared to that of unrelated children [24]. Studies tracking siblings of ASD children have detected vulnerabilities in some neurocognitive domains in the absence of an ASD diagnosis [72] suggesting that some of the NT siblings may exhibit a broader subclinical ASD phenotype.

Review of previous studies suggest that GI symptoms are more common in ASD children than in NT children, but these studies suffer from methodological differences in assessing GI symptoms [28–31]. Our assessment of GI symptoms differs from previous studies in that we used the international standard for assessing FGIDs in the general pediatric population. Because of potential overlaps between ROME III FGID diagnoses, we subdivided the ASD children and NT siblings each into two categories 1) those with at least one FGID and 2) those without any FGID. A high prevalence of children diagnosed with at least one FGID was observed in both the ASD (~40%) and NT siblings (~30%) in the current study. Previous epidemiological studies using the ROME III process to survey the prevalence of a child having at least one FGID in general pediatric clinics reported a prevalence of 2% and 16%, respectively [73,74], suggesting that NT siblings may also have a higher prevalence of FGID than the general pediatric population.

The current study detected an increased prevalence of functional constipation FGID in ASD children compared to NT siblings reached significance (see **Tables 1 and 2**). Constipation has been previously recognized as a significant comorbidity in ASD children [28–31]. Further studies on a larger sample of ASD and NT siblings (preferably family matched) as well as unrelated NT children will need to be conducted to determine 1) whether FGIDs, particularly functional constipation, are more prevalent in ASD children than their NT siblings and 2) whether FGIDs, particularly functional constipation, are more prevalent in NT siblings of ASD children than unrelated control children.

It has also been proposed that ASD children with GI symptoms may differ from ASD children without GI symptoms. Adams et al. [15] reported that GI symptoms, which were assessed by the modified 6-GSI score, correlated with ASD severity that was assessed by the parental completed Autism Treatment Evaluation Checklist (ATEC) [15]. However, in our study, the mean ADOS-CSS severity scores, which are based on physician rather than parental evaluations, were not significantly different between the ASD children with at least one FGID and those without any FGID.

To assess the overall emotional and behavioral status of ASD and NT siblings we analyzed parental completed CBCL6/18 normalized T scores. The results of this study demonstrated that ASD children with or without FGID exhibited significantly higher scores than the NT siblings without FGID, consistent with previous studies reporting higher scores in ASD children compared to control children [34–37]. However, this study demonstrated that the NT siblings with at least one FGID had normalized CBCL6/18 T-scores that approximated those of ASD children with or without at least one FGID, and were also significantly higher than NT siblings without any FGIDs.

Although this is one of the larger studies comparing ASD, FGID and the gut microbiota in ASD children and NT siblings, our sample size is still modest given the number of variables included. Recruitment of additional ASD children and their NT siblings (preferably family matched), as well as unrelated control children that use the same phenotyping tools will need to be conducted to confirm the findings reported in this study. Furthermore, it may be necessary to possibly restrict dietary intake of plant-derived supplements, such as chia seeds, to determine whether the Cyanobacteria/Chloroplast signal is of biological significance with respect to ASD pathophysiology.

## Supporting Information

S1 Fig Measurement platform comparison for MiSeq V1V2, MiSeq V1V3 and qPCR. The square root transformed relative abundance are plotted for each pair of platforms with the fitted line in proposed SEM model. For two perfectly consistent platforms, the fitted line is y = x.
(TIF)

S1 Table P-values for pairwise comparisons of CBCL scores (See Table 2) between ASD w FGID, ASD w/o FGID, NT sib w FGID, and NT sib w/o FGID. (DOCX)

S2 Table Relative abundances of major phyla for ASD children with FGID, ASD children without FGID, NT siblings with FGID, and NT siblings without FGID. The mean relative abundance (sequence count for phylum/total sequence count) ± standard deviation for the V1V2 and V1V3 datasets are listed below. The data from family matched and unmatched ASD children and NT siblings are included.
(DOCX)

S3 Table Relative abundances of low abundance phyla for ASD w FGID, ASD w/o FGID, NT w FGID, and NT w/o FGID, based on the V1V2 and V1V3 datasets. The mean relative abundance (sequence count for phylum/total sequence count) ± standard deviation are listed for each phylum. The data from family matched ASD children and NR siblings are included.
(DOCX)

S4 Table Relative abundance of selected taxa in ASD children with FGID, ASD w/o FGID, NT siblings with FGID and NT siblings without FGID. The mean relative abundances (sequence count for genera/total sequence count) ± standard deviations are listed for the V1V2 and the V1V3 datasets. N.D. is not detected based on filtering criteria. The taxa that exhibited significant effects of ASD, FGID and/or ASD*FGID first order interactions are **bolded**. The data from family matched and unmatched ASD children are included.
(DOCX)

S5 Table Measurement platform comparison for MiSeq V1V2, MiSeq V1V3 and qPCR. The 95% CI of estimated coefficients in the proposed SEM model are listed in a pairwise manner. For two perfectly consistent platforms, the slope is 1 and the intercept is 0.
(DOCX)
