# Supplementary figures and images for: Comparison of Fecal Microbiota in Children with Autism Spectrum Disorders and Neurotypical Siblings in the Simons Simplex Collection
Source: PLoS One. 2015 Oct 1;10(10):e0137725. doi: 10.1371/journal.pone.0137725 (PMC4591364; doi:10.1371/journal.pone.0137725)

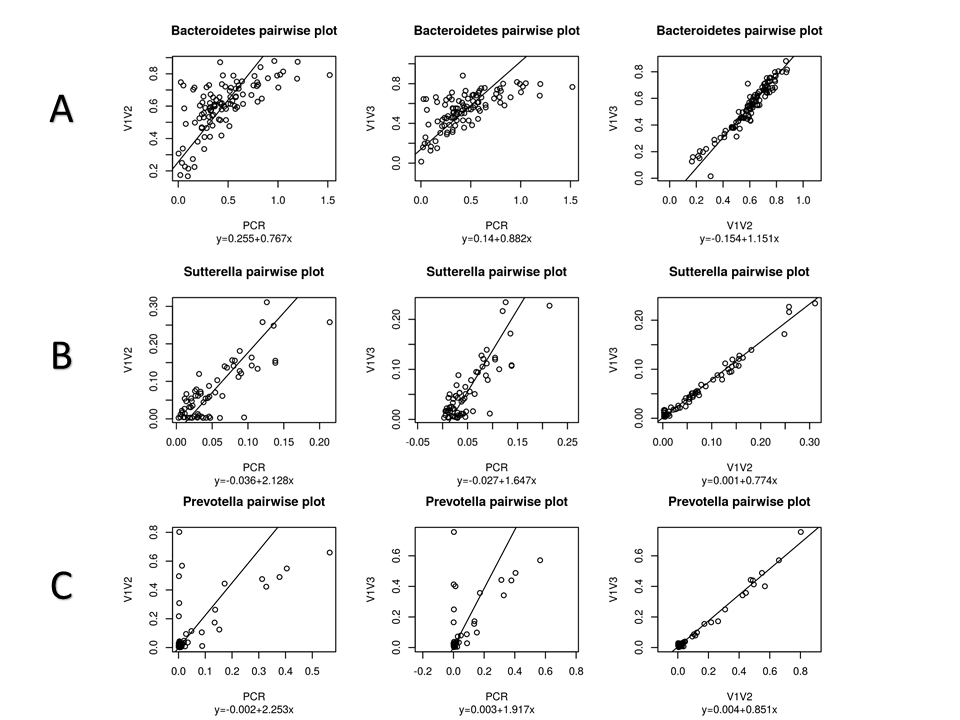

Supplement: S1 Fig — The square root transformed relative abundance are plotted for each pair of platforms with the fitted line in proposed SEM model. For two perfectly consistent platforms, the fitted line is y = x. (TIF) [file pone.0137725.s001.tif]
