# Supplementary material for: Comparison of Fecal Microbiota in Children with Autism Spectrum Disorders and Neurotypical Siblings in the Simons Simplex Collection
Source: PLoS One. 2015 Oct 1;10(10):e0137725. doi: 10.1371/journal.pone.0137725 (PMC4591364; doi:10.1371/journal.pone.0137725)
Supplement: S1 Table — (DOCX) [file pone.0137725.s002.docx]

**S1 Table. P-values for pairwise comparisons of CBCL scores (See Table 2) between ASD w FGID, ASD w/o FGID, NT sib w FGID, and NT sib w/o FGID.**

|  | ASD w FGID vs. ASD w/o FGID | ASD w FGID vs. NT sib w FGID | ASD w FGID vs. NT sib w/o FGID | ASD w/o FGID vs. NT sib w FGID | ASD w/o FGID vs. NT sib w/o FGID | NT sib w FGID vs. NT sib w/o FGID |
| --- | --- | --- | --- | --- | --- | --- |
| Total | 0.154 | 0.5855 | 0 | 0.3954 | 0 | 0 |
| Internal | 0.5502 | 0.3079 | 0.0003 | 0.1296 | 0.0005 | 0.0001 |
| External | 0.6682 | 0.7077 | 0.0002 | 0.5684 | 0.0017 | 0.0006 |
| Anxious/ Depressed | 0.2283 | 0.0023 | 0 | 0.0026 | 0 | 0.0913 |
| Withdrawn/ Depressed | 0.0759 | 0.0607 | 0.0001 | 0.0004 | 0.0219 | 0 |
| Somatic Complaints | 0.2573 | 0.1909 | 0.1163 | 0.0081 | 0.1827 | 0.0006 |
| Rule Breaking Behavior | 0.7822 | 0.2425 | 0.0004 | 0.2121 | 0.0014 | 0 |
| Aggressive Behavior | 0.3158 | 0.0166 | 0 | 0.0375 | 0 | 0.0713 |
| Social Problems | 0.0779 | 0.0049 | 0 | 0.1017 | 0.0003 | 0.0011 |
| Thought Problems | 0.3312 | 0.057 | 0 | 0.1527 | 0 | 0.0001 |
| Attention Problems | 0.154 | 0.5855 | 0 | 0.3954 | 0 | 0 |
