# Supplementary material for: Comparison of Fecal Microbiota in Children with Autism Spectrum Disorders and Neurotypical Siblings in the Simons Simplex Collection
Source: PLoS One. 2015 Oct 1;10(10):e0137725. doi: 10.1371/journal.pone.0137725 (PMC4591364; doi:10.1371/journal.pone.0137725)
Supplement: S2 Table — The mean relative abundance (sequence count for phylum/total sequence count) ± standard deviation for the V1V2 and V1V3 datasets are listed below. The data from family matched and unmatched ASD children and NT siblings are included. (DOCX) [file pone.0137725.s003.docx]

**S2 Table. Relative abundances of major phyla for ASD children with FGID, ASD children without FGID, NT siblings with FGID, and NT siblings without FGID.**  The mean relative abundance (sequence count for phylum/total sequence count) ± standard deviation for the V1V2 and V1V3 datasets are listed below. The data from family matched and unmatched ASD children and NT siblings are included.

| **Phylum (Major)** |  |  |
| --- | --- | --- |
| ***Firmicutes*** | **V1V2** | **V1V3** |
| ASD w FGID | 0.519 ± 0.156 | 0.564 ± 0.167 |
| ASD w/o FGID | 0.525 ± 0.182 | 0.570 ± 0.199 |
| NT w FGID | 0.547 ± 0.126 | 0.613 ± 0.134 |
| NT w/o FGID | 0.573 ± 0.179 | 0.628 ± 0.205 |
| ***Bacteroidetes*** | **V1V2** | **V1V3** |
| ASD w FGID | 0.397 ± 0.176 | 0.334 ± 0.166 |
| ASD w/o FGID | 0.395 ± 0.182 | 0.334 ± 0.189 |
| NT w FGID | 0.396 ± 0.138 | 0.315 ± 0.133 |
| NT w/o FGID | 0.361 ± 0.176 | 0.293 ± 0.186 |
| ***Actinobacteria*** | **V1V2** | **V1V3** |
| ASD w FGID | 0.035 ± 0.047 | 0.026 ± 0.037 |
| ASD w/o FGID | 0.029 ± 0.039 | 0.028 ± 0.047 |
| NT w FGID | 0.022 ± 0.018 | 0.018 ± 0.016 |
| NT w/o FGID | 0.026 ± 0.034 | 0.020 ± 0.031 |
| ***Proteobacteria*** | **V1V2** | **V1V3** |
| ASD w FGID | 0.020 ± 0.028 | 0.012 ± 0.018 |
| ASD w/o FGID | 0.020 ± 0.018 | 0.010 ± 0.008 |
| NT w FGID | 0.008 ± 0.007 | 0.005 ± 0.004 |
| NT w/o FGID | 0.021 ± 0.018 | 0.021 ± 0.018 |
