# Supplementary material for: Comparison of Fecal Microbiota in Children with Autism Spectrum Disorders and Neurotypical Siblings in the Simons Simplex Collection
Source: PLoS One. 2015 Oct 1;10(10):e0137725. doi: 10.1371/journal.pone.0137725 (PMC4591364; doi:10.1371/journal.pone.0137725)
Supplement: S3 Table — The mean relative abundance (sequence count for phylum/total sequence count) ± standard deviation are listed for each phylum. The data from family matched ASD children and NR siblings are included. (DOCX) [file pone.0137725.s004.docx]

**S3 Table. Relative abundances of low abundance phyla for ASD w FGID, ASD w/o FGID, NT w FGID, and NT w/o FGID, based on the V1V2 and V1V3 datasets.**  The mean relative abundance (sequence count for phylum/total sequence count) ± standard deviation are listed for each phylum. The data from family matched ASD children and NR siblings are included.

| **Phylum (Low Abundance)** |  |  |
| --- | --- | --- |
| ***RF3*** | **V1V2** | **V1V3** |
| ASD w FGID | 26 ± 77E-4 | 37 ± 11E-4 |
| ASD w/o FGID | 8 ± 31E-4 | 11 ± 45E-4 |
| NT w FGID | 0 ± 0E-4 | 0 ± 0E-4 |
| NT w/o FGID | 3 ± 9E-4 | 5 ± 16E-4 |
| ***Tenericutes*** | **V1V2** | **V1V3** |
| ASD w FGID | 15 ± 68E-4 | 4 ± 18E-4 |
| ASD w/o FGID | 19 ± 110E-4 | 12 ± 70E-4 |
| NT w FGID | 8 ± 22E-4 | 3 ± 10E-4 |
| NT w/o FGID | 5 ± 15E-4 | 1 ± 1E-4 |
| ***Cyanobacteria*** | **V1V2** | **V1V3** |
| ASD w FGID | 8 ± 21E-4 | 11 ± 36E-4 |
| ASD w/o FGID | 20 ± 113E-4 | 14 ± 20E-4 |
| NT w FGID | 0 ± 0E-4 | 0 ± 0E-4 |
| NT w/o FGID | 2 ± 9E-4 | 2 ± 7E-4 |
| ***Verrucomicrobia*** | **V1V2** | **V1V3** |
| ASD w FGID | 1 ± 2E-4 | 110 ± 150E-4 |
| ASD w/o FGID | 1 ± 2E-4 | 102 ± 195E-4 |
| NT w FGID | 1 ± 3E-4 | 71 ± 143E-4 |
| NT w/o FGID | 0 ± 1E-4 | 34 ± 62E-4 |
| ***Lentisphaerae*** | **V1V2** | **V1V3** |
| ASD w FGID | 1 ± 2E-4 | 10 ± 21E-4 |
| ASD w/o FGID | 0 ± 0E-4 | 1 ± 5E-4 |
| NT w FGID | 1 ±2E-4 | 6 ± 21E-4 |
| NT w/o FGID | 0 ±1E-4 | 4 ±16E-4 |
| ***Fusobacterium*** | **V1V2** | **V1V3** |
| ASD w FGID | 1 ± 3E-4 | 2 ± 7E-4 |
| ASD w/o FGID | 0 ± 1E-4 | 0 ± 1E-4 |
| NT w FGID | 0 ± 0E-4 | 0 ± 0E-4 |
| NT w/o FGID | 0 ± 1E-4 | 0 ± 1E-4 |
