# Supplementary material for: Comparison of Fecal Microbiota in Children with Autism Spectrum Disorders and Neurotypical Siblings in the Simons Simplex Collection
Source: PLoS One. 2015 Oct 1;10(10):e0137725. doi: 10.1371/journal.pone.0137725 (PMC4591364; doi:10.1371/journal.pone.0137725)
Supplement: S4 Table — The mean relative abundances (sequence count for genera/total sequence count) ± standard deviations are listed for the V1V2 and the V1V3 datasets. N.D. is not detected based on filtering criteria. The taxa that exhibited significant effects of ASD, FGID and/or ASD*FGID first order interactions are bolded. The data from family matched and unmatched ASD children are included. (DOCX) [file pone.0137725.s005.docx]

**S4 Table. Relative abundance of selected taxa in ASD children with FGID, ASD w/o FGID, NT siblings with FGID and NT siblings without FGID.** The mean relative abundances (sequence count for genera/total sequence count) ± standard deviations are listed for the V1V2 and the V1V3 datasets. N.D. is not detected based on filtering criteria. The taxa that exhibited significant effects of ASD, FGID and/or ASD*FGID first order interactions are **bolded**. The data from family matched and unmatched ASD children are included.

| **Phylum/Taxon** |  |  |
| --- | --- | --- |
| ***Cyanobacteria/Chloroplast*** | **V1V2** | **V1V3** |
| ASD w FGID | 650 ± 2111E-6 | 1024 ± 3627E-6 |
| ASD w/o FGID | 6 ± 13E-6 | 5 ± 13E-6 |
| NT w FGID | 10 ± 12E-6 | 4 ± 7E-6 |
| NT w/o FGID | 13 ± 29E-6 | 8 ± 31E-6 |
| ***Firmicutes/Asteroleplasma*** | **V1V2** | **V1V3** |
| ASD w FGID | 183 ± 618E-6 | 195 ± 667E-6 |
| ASD w/o FGID | 54 ± 265E-6 | 63 ± 302E-6 |
| NT w FGID | 2 ± 7E-6 | 3 ± 12E-6 |
| NT w/o FGID | 163 ± 597 E-6 | 138 ± 476E-6 |
| ***Proteobacteria/Thalassospira*** | **V1V2** | **V1V3** |
| ASD w FGID | 1914 ± 4669E-6 | 2075 ± 5381E-6 |
| ASD w/o FGID | 590 ± 2354E-6 | 701 ± 2967E-6 |
| NT w FGID | 475 ± 1691E-6 | 558 ± 1768E-6 |
| NT w/o FGID | 1092 ± 2458E-6 | 1769 ± 4254E-6 |
| ***Proteobacteria/Burkholderia*** | **V1V2** | **V1V3** |
| ASD w FGID | 6 ± 29E-6 | 2 ± 6E-6 |
| ASD w/o FGID | 6 ± 34E-6 | 0 ± 1E-6 |
| NT w FGID | 0 ± 0E-6 | 0 ± 0E-6 |
| NT w/o FGID | 6 ± 29E-6 | 0 ± 1E-6 |
| ***Proteobacteria/Comamonadaceae*** | **V1V2** | **V1V3** |
| ASD w FGID | 45 ± 182E-6 | 28 ± 109E-6 |
| ASD w/o FGID | 19 ± 68E-6 | 1 ± 3E-6 |
| NT w FGID | 10 ± 14E-6 | 2 ± 5E-6 |
| NT w/o FGID | 17 ± 39E-6 | 23 ± 85E-6 |
| ***Fusobacteria/Fusobacteriales*** | **V1V2** | **V1V3** |
| ASD w FGID | 33 ± 159E-6 | 72 ± 358E-6 |
| ASD w/o FGID | 11 ± 62E-6 | 1 ± 4E-6 |
| NT w FGID | 3 ± 5E-6 | 5 ± 19E-6 |
| NT w/o FGID | 18 ± 79E-6 | 10 ± 40E-6 |
| ***Bacteroidetes/Prevotellaceae*** | **V1V2** | **V1V3** |
| ASD w FGID | 1315 ± 6517E-6 | 745 ± 3716E-6 |
| ASD w/o FGID | 62 ± 202E-6 | 1 ± 3E-6 |
| NT w FGID | 16 ± 34E-6 | 10 ± 22E-6 |
| NT w/o FGID | 250±873E-6 | 2 ± 6E-6 |
| ***Actinobacteria/Mobiluncus*** | **V1V2** | **V1V3** |
| ASD w FGID | N.D. | 3 ± 12E-6 |
| ASD w/o FGID | N.D. | 38 ± 219E-6 |
| NT w FGID | N.D. | 2 ± 6E-6 |
| NT w/o FGID | N.D. | 1 ± 3E-6 |
| *Proteobacteria/Sutterella* | **V1V2** | **V1V3** |
| ASD w FGID | 0.0076 ± 0.023 | 0.048 ± 0.014 |
| ASD w/o FGID | 0.0063 ± 0.010 | 0.037 ± 0.06 |
| NT w FGID | 0.0036 ± 0.007 | 0.022 ± 0.04 |
| NT w/o FGID | 0.0076 ± 0.017 | 0.045 ± 0.01 |
| *Bacteroidetes/Prevotella* | **V1V2** | **V1V3** |
| ASD w FGID | 0.023 ± 0.088 | 0.017 ± 0.066 |
| ASD w/o FGID | 0.027 ± 0.080 | 0.017 ± 0.052 |
| NT w FGID | 0.069 ± 0.180 | 0.056 ± 0.156 |
| NT w/o FGID | 0.024 ± 0.071 | 0.019 ± 0.060 |
