# Supplementary material for: Comparison of Fecal Microbiota in Children with Autism Spectrum Disorders and Neurotypical Siblings in the Simons Simplex Collection
Source: PLoS One. 2015 Oct 1;10(10):e0137725. doi: 10.1371/journal.pone.0137725 (PMC4591364; doi:10.1371/journal.pone.0137725)
Supplement: S5 Table — The 95% CI of estimated coefficients in the proposed SEM model are listed in a pairwise manner. For two perfectly consistent platforms, the slope is 1 and the intercept is 0. (DOCX) [file pone.0137725.s006.docx]

**S5 Table. Measurement platform comparison for MiSeq V1V2, MiSeq V1V3 and qPCR.** The 95% CI of estimated coefficients in the proposed SEM model are listed in a pairwise manner. For two perfectly consistent platforms, the slope is 1 and the intercept is 0.

| **Sutterella** | **95% CI of intercept** | **95% CI of slope** |
| --- | --- | --- |
| V1V2~PCR | (-0.039,-0.033) | (2.08,2.18) |
| V1V3~PCR | (-0.031,-0.023) | (1.57,1.73) |
| V1V3~V1V2 | (-0.003,0.005) | (0.71,0.84) |
|  |  |  |
| **Prevotella** | **95% CI of intercept** | **95% CI of slope** |
| V1V2~PCR | (-0.003,0) | (1.71,2.30) |
| V1V3~PCR | (0.001,0.005) | (1.86,1.97) |
| V1V3~V1V2 | (0.001,0.007) | (0.81,0.89) |
|  |  |  |
| **Bacteriodetes** | **95% CI of intercept** | **95% CI of slope** |
| V1V2~PCR | (0.181,0.329) | (0.60,0.93) |
| V1V3~PCR | (0.054,0.226) | (0.69,1.07) |
| V1V3~V1V2 | (-0.212,-0.096) | (1.05,1.25) |
